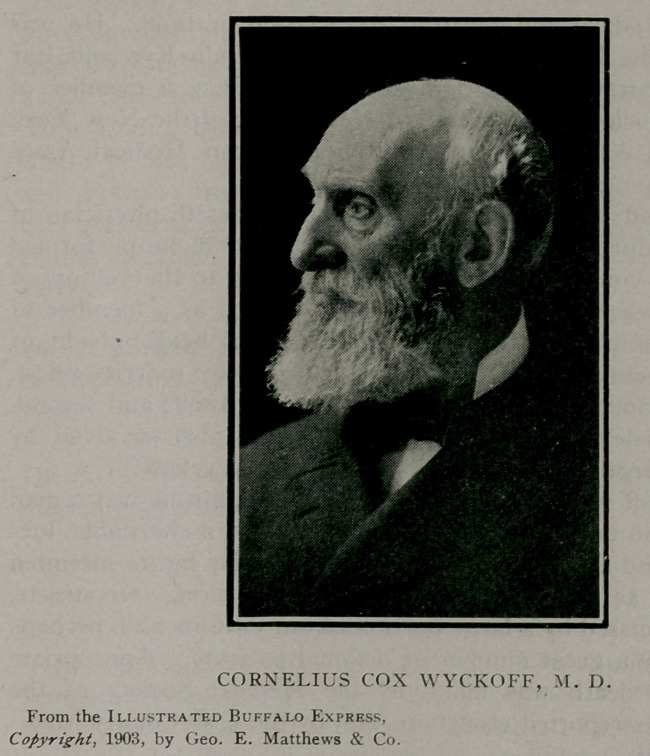# Dr. Cornelius Cox Wyckoff

**Published:** 1903-12

**Authors:** 


### OBITUARY.

Dr. Cornelius Cox Wyckoff, of Buffalo, died at his residence, 482 Delaware Avenue, Saturday evening, November 7,

CORNELIUS COX WYCKOFF, M. D.

From the Illustrated Buffalo Express,
*Copyright*, 1903, by Geo. E. Matthews & Co.

1903, aged 81 years. His death was unexpected, though his health had been somewhat impaired of late. Notwithstanding this fact, he continued his professional occupation until the day before his death. He was born at Romulus, N. Y., August 5, 1822, and received his preliminary education at Genesee Wesleyan Seminary, Lima, N. Y., after which he began the study of medicine and entered Geneva Medical College. When the latter school was discontinued, Mr. Wyckoff was among the students who came to Buffalo, and he received his doctorate degree from the University of Buffalo at its second annual commencement, held June 14, 1848. Dr. Wyckoff immediately after graduation began the practice of his profession in this city, and pursued an active medical career until his death, covering a period of fifty-five years. It comes to few men to live so long in one place engaged constantly in professional occupation.

Dr. Wyckoff became a member of the visiting staff of the Buffalo General Hospital when it was organised in 1858, continuing to serve either as attending or consulting physician until the time of his death, and was the last surviving member of the original staff. He joined the Medical Society of the County of Erie in 1849, and served as president in 1864; and he was president of the Buffalo Medical and Surgical Association in 1858. He was president of the board of trustees of the Buffalo Eye and Ear Infirmary, a curator of the University of Buffalo, a member of the Medical Society of the State of New York, of the New York State Medical Association, and of the American Medical Association.

Dr. Wyckoff in his earlier years served as health physician of Buffalo, and during the cholera epidemic of 1852 he performed masterful and courageous service in ministering to the victims of that dread disease. In his later years he served as a member of the park commission, the only office he ever held outside of strictly professional lines. Dr. Wyckoff was twice married; first, in 1849, to Frances Hall Hastings, who died in 1869; and second, to Alice Lindsley Hall who survives. He is also survived by two sons, George S. and Cornelius Hastings Wyckoff.

Dr. Wyckoff was not only an able physician, but he was a man of refinement in taste and cultivation in manners, a charitable, loving, loyal friend and useful citizen. His familiar figure mounted on horseback, as was his daily custom, riding along our streets, will be sadly missed by a large portion of our citizens and, perhaps most of all, by a great number of devoted patients. Appropriate action on his death was taken by the Medical Society of the County of Erie, reported elsewhere, and by the Erie County Medical Association.

## Figures and Tables

**Figure f1:**